# Evaluation of Hepatoprotective Effect of *Acantholimon Gilliati *Eerial Part Methanolic Extract

**Published:** 2017

**Authors:** Rouhollah Gazor, Mehrdad Asgari, Ardalan Pasdaran, Fahimeh Mohammadghasemi, Ebrahim Nasiri, Zahra Atrkar Roushan

**Affiliations:** a *Cellular and Molecular Research Center, Guilan University of Medical Sciences, Rasht, Iran. *; b *Student Research Center, Guilan University of Medical Sciences, Rasht, Iran. *; c *Research and development center of plants and medicinal chemistry, Guilan University of Medical Sciences, Rasht, Iran. *; d *Medicinal Plants Processing Research Center, Shiraz University of Medical Sciences, Shiraz, Iran. *; e *Department of Anatomical Sciences, Faculty of Medicine, Guilan University of Medical Sciences, Rasht, Iran.*; f *Department of social Medicine, Faculty of Medicine, Guilan University of Medical Sciences, Rasht, Iran.*

**Keywords:** Plumbaginaceae, *Acantholimon gilliati*, methanolic extract, hepatoprotective effect, formaldehyde induced liver injury

## Abstract

The aerial parts of *Acantholimon gilliati* was extracted by n-hexane, dichloromethane and methanol. Methanolic extract tested for hepatoprotective effects on formaldehyde liver injury in mice. The maximum effect that the methanolic extract showed protective effect on this experiment against formaldehyde observed in 5 and 10 mg. Also other concentrations of this extract showed positive effect compared to toxicant on morphology and biochemical factors of the liver. Results showed that the methanolic extract of the *A. gilliati *has a protective include functional and enzymatic stablingeffect on liver.

## Introduction

The liver is vital organ in the maintenance of homeostasis and exogenous detoxification, responsible for multiple metabolic functions and physiological processes such as bile production, energy generation, vitamin storage, metabolism of carbohydrates, proteins and lipids (1). With regard to these functions, hepatic injuries are the principal threats to public health and one of the primary cause of morbidity and mortality in worldwide (2). Formaldehyde (FA) is a monocarbon, flammable gas with suffocating odour that released from many environmental sources. Abundantly used in many industries such as chemicals, medicinal and cosmeceutical. Formaldehyde can react to key cellular components such as DNA, proteins and lipids. The hepatotoxicity of FA has been shown in previous studies (3, 4).While such detoxification leading to liver goes under stress but there are some medications recommended for hepatoprotection that most of them are no completely effective drugs for stimulating hepatic function or aid in regenerating hepatic cells. Thus, need of the natural based novel therapeutic with more effective clearly necessary. The hepatoprotective activity of many terrestrial plants such as grape vine, Turmeric, chicory and others popularized (5, 6). Clinical studies also demonstrated efficacy and safety of a number of herbal products in the treatment of liver diseases (7).

*Acantholimon gilliati* (Plumbaginaceae) is a woody shrub, highly branched, with purple flowers that widely distributed in many regions of Iran which known as "Kolah Mir-hassan ". It is being used to treat diabetes and known for hepatoprotective effects in the west region of Iran. 

The present study focused on evaluating the potential hepatoprotective effects of methanolic extracts (ME) from *A.gilliati* on formaldehyde (FA) induced liver injury in mice.

## Experimental

*Plant*

*A.gilliati *were collected at the flowering period from wild population growing in East Azarbaijan province, Iran. Voucher specimens were authenticated by the Pharmacognosy Department and voucher specimens, Nos 2558, deposited in the herbarium of Pharmacognosy department of pharmacy faculty of Guilan University of medical sciences, Rasht, Iran. 

***Experimental animals***

Fifty six adult male albino mice (20-25g) obtained used for this investigation, source of these animal was Razi vaccine and serum research institute. They were housed in animal care facility with 12 h light and dark cycle (25 ± 2 ºC, 60%-70% humidity). All welfare and experimental animal procedures carried on the US National Institutes of Health (NIH publication no. 85–23, revised 1996) guideline for the care and use of laboratory animals with consideration Guilan University of Medical Science published guidelines on the use and care of laboratory animals.

***Preparation of plant extracts***

300 g of powdered aerial parts of *A.gilliati* was used for extraction with 2.5 L of n-hexane, dichloromethane and methanol in dried by continues extraction methods by Soxhlet apparatuses during 72 h period for each solvent. All extracts were filtered and concentrated by using vacuum rotary evaporator at 40 °C.After solvent evaporation, methanolic extract was dried by using an oven at 40 °C for 4-5 h. The yield of the methanolic extract was 35 g from 100 g of aerial partsof plant. 2g of this methanolic extract was used for next step of hepatoprotective activity test in this experiment.

*Experimental design*

This investigation designed based on previous Gulec *et al*. method (8). In this method eight groups of laboratory animals were used (seven mice ineach group). Group I (E0 normal control) received normal saline. Group II (E1 toxicant control) received only 37% formaldehyde (10 mg/kg) for two weeks (3 days a week).Groups III-VIII (E2-E7) were subjected to 37% formaldehyde (10 mg/kg), after a hour these groups treated with methanolic extracts (ME) intraperitoneally at a dose of 5, 10, 15, 20, 50 and 100 mg, respectively for 2 weeks (once every other day). The animals were scarified by cervical dislocation after the ether anesthetisa on the 15th day. Blood was collected by cardiac and IVC puncture and allowed to clot, for serum separation used centrifuge with 15000 rpm for 15 min. After serum separation, samples collected and kept at 4ºC for futureexaminations. 10% formalin used for tissues fixation for future histopathological assessment.

*Determination of biochemical parameters*

The serum alkaline phosphatase (ALP), aspartate aminotransferase (AST) alanine aminotransferase (ALT) were assayed according to standard methods. For determination of enzymes activities were used photometric method based on lactate dehydrogenase (DGKC) of conversion P-Nitrophenylphosphate+H_2_O to phosphate + *p*-Nitrophenol (assay kits Parsazmun, Iran).

*Histopathological analysis*

Removed livers were fixed by using of 10% buffered formalin for 72 h, after the dehydration process and placed in paraffin block.For tissues slide preparation 5 μm sections made from tissues-paraffin block. This tissues slide stained with haematoxylin–eosin for future histological assessments. As histological damages characters, we considered several histological parameters as criteria for tissues damage estimating include congested blood vessel, congested blood sinusoids, degeneration of lobules, inflammatory cell, necrotic cells, and apoptotic cells, and observed damages was graded based on previous works from scores 0-3 (9-11).

*Statistical analysis*

Results from *in-vivo* tests were performed by one-way analysis of variance (ANOVA) followed post Hoc (Tukey) to detect inter group differences where Value of *p*< 0.05 was considered to be significant. All data were expressed as mean ± SD. 

## Results

*Effects of methanolic extract (ME) on hepatic markers*

**Figure 1:**
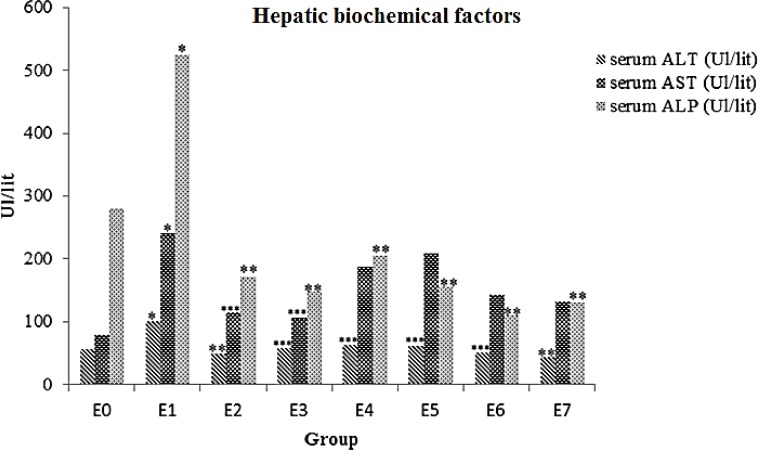
Effects of ME on serum levels of ALT, AST and ALP in FA- intoxicated mice. Data are expressed as the mean ± SD, n = 7. * p= 0.002, compared to the control group, ** p < 0.001 and *** p < 0.005 compared to the FA group. E0 group: normal saline; E1 group: 10mg/kg FA; E2 group: 5 mg ME + FA; E3 group: 10 mg ME + FA; E4 group: 15 mg ME + FA; E5 group: 20 mg ME + FA; E6 group: 50 mg ME + FA; E7 group: 100 mg ME + FA

Results showed that animals ALT, AST and ALP enzymes levels signiﬁcantly increased in E1 group (*p*= 0.002). All animals that received ME displayed remarkable decrease in ALT and ALP level compared to the formalin group (p < 0.05).Strong hepatoprotective effect of ME observed in E2 (5 mg) and E3 (10 mg) groups (p < 0.05) against E1 group (Table 1, Figure 1). 

**Table 1:** The liver enzymes alteration after treatment with methanolic extracts of *A.gilliati* in FA induced liver injury mice

Groups	Dosage	ALT(Ul/lit)	AST(Ul/lit)	ALP(Ul/lit)
E0	normal saline 0.9%	56.71±28.62	79.14±22.61	279.57±194.74
E1	10mg/kg FA 37%	100±5.09^a^	240.57±47.46^a^	523.86±124.08^a^
E2	10mg/kg FA+5mg ME	49.43±14.08^b^	115±81.50^c^	171.57±85.64^b^
E3	10mg/kg FA+10mg ME	58.86±13.33^c^	105.14±29.96^c^	147.71±30.45 b
E4	10mg/kg FA+15mg ME	63±10.49^c^	187.60±87.23	204.80±102.60 b
E5	10mg/kg FA+20mg ME	61.60±34.13^c^	209.40±152.06	154.80±25.37 b
E6	10mg/kg FA+50mg ME	50.60±10.47^c^	143.20±25.47	114.40±11.95 b
E7	10mg/kg FA+100mg ME	43.60±12.78^b^	132.80±41.40	131.40±46.53 b

Data are shown as the mean ± SD, n = 7.

ALT: alanine aminotransferase, AST: aspartate aminotransferase, ALP:alkaline phosphatase

a : p= 0.002, in compared with control

b : p < 0.001, in compared with FA group

c : p < 0.005. in compared with FA group

**Histopathology**

**Figure 2:**
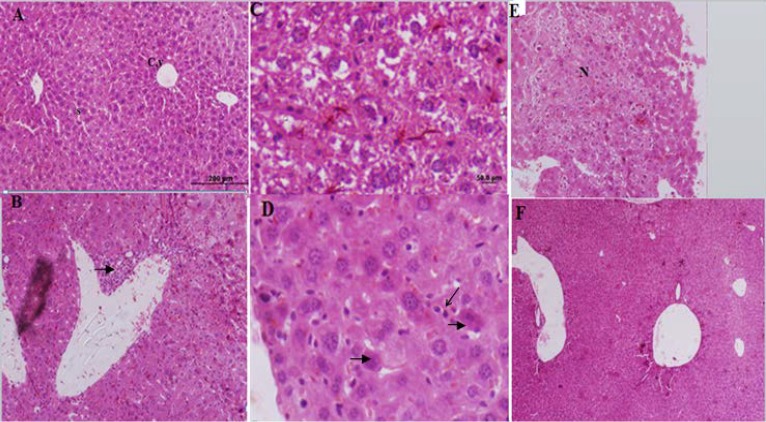
Histological figure from a mice liver (A): showed a normal hepatocyte in control group. X100 (B, C, D and E): liver sections in FA treated groups (Group E1) visible inﬁltration of inﬂammatory cells, hepatocytes degenerations, increased kupffer cells, hypereosinophilic cytoplasm and necrosis. (F): Improved liver changes in E2 group

**Figure 3:**
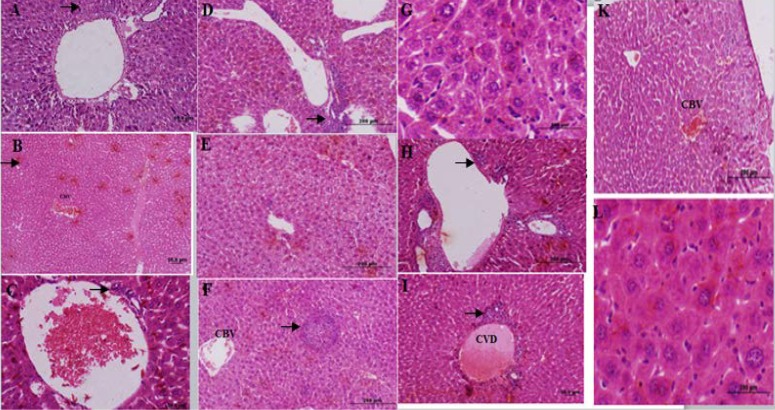
Histological figure from a mice liver (A): lymphocyte inﬁltration observed in E3 group. (B): congested blood vessel (CBV) and lymphocyte inﬁltration in E4 group. (C): hepatocyte view of E5 group central with venous distension, and mild lymphocyte inﬁltration (D-G): E6 group hepatocytes view with lymphocytes accumulation (D), vacuole formation (E), lymphocytes inﬁltration (F) and kupffer cells accumulation (G). (H-L): liver sections in FA treated group (E7 group) lymphocytes accumulation (H), central venous distension (I), congested blood vessel (K) and kupffer cells accumulation (L).

The control group liver tissues showed normal cellular pattern including recognizable hepatic cells with normal central vein and sinusoidal region (Figure 2A). In contrast, liver tissues in the formalin group (E1) (10 mg formaldehyde 37%) showed the most prevalent damage between all groups. In the liver tissues sections of E1 group were detected congested sinusoids, congested vessel, and inﬁltration of inflamed cells (lymphocytes), necrotic sections in addition to increased hypereosinophilic cytoplasm, and vacuolated hepatocytes that identified massive liver injuries (Figure 2B-E). Receiving 5 mg ME in E2 group almost ameliorated formaldehyde 37% effects on liver tissues. In microscopic view liver architecture was more similar to the control group (Figure 2F). The mice liver sections of E3group (treated with ME 10 mg, Figure 3A) showed a relatively normal hepatic pattern, however a mild inﬁltration of lymphocyte cell predictable compared to E1 group.E4 group (10 mg/kg formaldehyde 37% and 15 mg ME) liver sections showed a relatively normal pattern of sinusoids and congested vessel with a mild lymphocyte aggregation (Figure 3B). Mice treated with 20 mg of the ME in E5 group exhibited some histopathology improvement in the liver, although same venal changes like E4 group observable in liver samples (Figure 3C). In final group (E6) 50 mg administration of ME caused some destructive changes in liver foundation such as aggregate of vacuolated kupffer cells, lymphocytes aggregation, and congested blood vessel (Figure 3 D-G). Liver histological analyses of E7 group exhibited the same E6 group injuries pattern include mononuclear cells infiltration, kupffer cells aggregation, and central venous distension (Figure 2 H-L). Based on these liver tissues histological evidences conﬁrmed that *A.gilliati *methanolic extract can be effective against formalin hepatotoxicity in 5 and 10 mg/ kg.

## Discussion

According to the pervious investigation indicated that exposing to the formaldehyde for 2 weeks can be induced rough in liver functions and cells. Based on our finding using *A.gilliati *methanolic extract can be ameliorated the FA liver injury especially in lower doses (5 and 10 mg/ kg). As basic scheme of liver tissues FA injuries can be noted the inﬂammatory cells, hypereosinophilic cytoplasm, activated kupffer cells aggregation, vacuolated hepatocytes, and congested sinusoids(12, 13). Aggregation of the von kupffer cells in some hepatic lobules of E1 group probably was attributed to accumulated formaldehyde, which led to the denaturation of protein molecules. The other possible mechanisms of the hepatotoxicity of FA include increasing production of reactive oxygen species (ROS), microvesicular steatosis and DNA-protein cross links (DPC) (14-16).

During to formaldehyde exposure into the tissues oxidative damage resulting from active oxygen species has been detected. Previous studies showed that FA with chorionic exposure and low concentrations can be triggered oxidative stress streams. These active oxidant species can caused develop hepatotoxicity through the oxidative reaction on cellular important compartments include DNA, cell membrane lipids, and cellular proteins (17, 18). Investigations indicated that formaldehyde exposure can be decreased the superoxide dismutase and glutathione peroxidas activities, also same decreasing exhibited in necrosis factor kB (NF-kB) and activator protein 1 activities(19). During to the liver formaldehy detoxification, hepatic enzymes released into the blood and caused an elevated levels of serum enzymes alanine aminotransferase (*ALT*)and aspartate aminotransferase (*AST*). This ALT and AST elevation known as indicative cellular and functional damages of the liver (20).

Natural products as hepatoprotective remedies have the long history between many nations, this therapeutic potentials originated from various phytochemical compounds class such as terpenoids, flavonoids glycosides, iridoids glycosides and many others compounds (21, 22). Maximum hepatoprotective effects of *A.gilliati *methanolic extract can be exhibited in 10 mg of this extract. Although same effect observed in other concentration against formaldehyde. According to the exhibited response values, the experimental factors that evidented from the healed treated test groups and formaldehyde groups could be explained a quadratic interactions dose dependent manner for *A.gilliati *methanolic extract hepatoprotective potential. A one probably hepatoprotective activity mechanism of *A.gilliati *methanolic extract against the formaldehyde could be resulted by antioxidant effect against very active species intermediates induced by formaldehyde.

## Conclusion

Lipid peroxidation induced by formaldehyde and other free radical intermediates has been considered as one of the main causes of the liver toxicity of this compound. Therefore, inhibition production of these radicals could be considered as important mechanism of *A.gilliati *methanolic extract protection against formaldehyde liver injury. The results of this study indicate that the areal parts of *A.gilliati *methanolic extract hepatoprotective effect against formaldehyde induced liver damage is very distinctive. ALT and AST serum levels back towards in normal value (at the 5 and 10 mg dose of ME) that this probably indicate plasma membrane stabilization of hepatic cellular systems. In all histopathological views can be observed hepatoprotective support of *A.gilliati *methanolic extract on hepatocellular foundations.
